# Retraction Note: FK506-binding protein-5 in high-fat diet-induced metabolic dysfunction-associated steatotic liver disease

**DOI:** 10.1038/s41598-026-69443-0

**Published:** 2026-09-02

**Authors:** Li-Ling Wu, Yu-Jen Liao, Wei-Hao Peng, Luen-Kui Chen, Yi-Chen Huang, Chia-Yen Chen, Chi-Chang Juan

**Affiliations:** 1 Department and Institute of Physiology, National Yang Ming Chiao Tung University, Taipei, 11221 Taiwan; 2 School of Medicine, National Tsing Hua University, Hsinchu, 300044 Taiwan R.O.C.; 3 Health Innovation Center, National Yang Ming Chiao Tung University, Taipei, Taiwan; 4 Microbiota Research Center, National Yang Ming Chiao Tung University, Taipei, Taiwan

Retraction of: *Scientific Reports* https://doi.org/10.1038/s41598-026-38549-w, published online 16 February 2026

The Editors have retracted this Article.

After publication, concerns were raised that the description of the methods and the data does not match the publicly deposited dataset from this study. Further concerns were raised about substantial textual overlaps with previously published papers, most significantly the description of results which appears to be taken from another study on a different topic^1^ with no common authors. The Editors therefore no longer have confidence in the contents of this Article.

Luen-Kui Chen and Chi-Chang Juan agree with the retraction and its wording. Li-Ling Wu disagrees with the retraction. Yu-Jen Liao, Wei-Hao Peng, Yi-Chen Huang, and Chia-Yen Chen did not respond to the Editors' correspondence about the retraction.
